# Short day transcriptomic programming during induction of dormancy in grapevine

**DOI:** 10.3389/fpls.2015.00834

**Published:** 2015-11-04

**Authors:** Anne Y. Fennell, Karen A. Schlauch, Satyanarayana Gouthu, Laurent G. Deluc, Vedbar Khadka, Lekha Sreekantan, Jerome Grimplet, Grant R. Cramer, Katherine L. Mathiason

**Affiliations:** ^1^Northern Plains BioStress Laboratory, Plant Science Department, South Dakota State UniversityBrookings, SD, USA; ^2^Department of Biochemistry and Molecular Biology, University of Nevada, RenoReno, NV, USA; ^3^Department of Horticulture, Oregon State UniversityCorvallis, OR, USA; ^4^Instituto de Ciencias de la Vid y del Vino (CSIC, Universidad de La Rioja, Gobierno de La Rioja)Logroño, Spain

**Keywords:** *Vitis riparia*, Seyval, bud, VitisNet, ABA, resveratrol, trehalose, raffinose

## Abstract

Bud dormancy in grapevine is an adaptive strategy for the survival of drought, high and low temperatures and freeze dehydration stress that limit the range of cultivar adaptation. Therefore, development of a comprehensive understanding of the biological mechanisms involved in bud dormancy is needed to promote advances in selection and breeding, and to develop improved cultural practices for existing grape cultivars. The seasonally indeterminate grapevine, which continuously develops compound axillary buds during the growing season, provides an excellent system for dissecting dormancy, because the grapevine does not transition through terminal bud development prior to dormancy. This study used gene expression patterns and targeted metabolite analysis of two grapevine genotypes that are short photoperiod responsive (*Vitis riparia*) and non-responsive (*V. hybrid*, Seyval) for dormancy development to determine differences between bud maturation and dormancy commitment. Grapevine gene expression and metabolites were monitored at seven time points under long (LD, 15 h) and short (SD, 13 h) day treatments. The use of age-matched buds and a small (2 h) photoperiod difference minimized developmental differences and allowed us to separate general photoperiod from dormancy specific gene responses. Gene expression profiles indicated three distinct phases (perception, induction and dormancy) in SD-induced dormancy development in *V. riparia*. Different genes from the NAC DOMAIN CONTAINING PROTEIN 19 and WRKY families of transcription factors were differentially expressed in each phase of dormancy. Metabolite and transcriptome analyses indicated ABA, trehalose, raffinose and resveratrol compounds have a potential role in dormancy commitment. Finally, a comparison between *V. riparia* compound axillary bud dormancy and dormancy responses in other species emphasized the relationship between dormancy and the expression of *RESVERATROL SYNTHASE* and genes associated with C3HC4-TYPE RING FINGER and NAC DOMAIN CONTAINING PROTEIN 19 transcription factors.

## Introduction

Wild grapevine species (*Vitaceae*) are predominately native to the northern hemisphere; however, the production of grape cultivars is widely distributed, and the grapevine is one of the temperate fruit crops most frequently damaged by winter freezing temperatures. Development of dormancy allows the grapevine to better tolerate the stress of unfavorable winter temperatures, but may limit the production range (Fennell, 2004). Indeed, winter sub-zero and chilling temperatures limit the production range of many cultivars, and as climate changes regional microenvironments, they are subjected to increasingly variable dormancy, acclimation and spring bud break conditions. Therefore, it is necessary to have a better understanding of the mechanisms involved in dormancy. This will allow us to match cultivars appropriately with growing sites, improve cultural practices that minimize freezing injury, and aid in breeding and selecting grapevines for sustained winter survival.

In many temperate woody plants, growth cessation and dormancy development are necessary for acclimation to occur, and a decreasing day length promotes the transition to a winter tolerant state. For example, short days (SD) induces growth cessation, terminal bud set and dormancy in birch, chestnut, oak, peach, and poplar trees. Dormancy results from multiple sequential suites of gene expression from early perception, terminal bud set and finally transition into dormancy (Ruttink et al., 2007; Jiménez et al., 2010; Santamaría et al., 2011; Ueno et al., 2013). Over-expression of *ABSCISIC ACID INSENSITIVE3 (ABI3), FLOWERING TIME (FT)* or *PHYTOCHROME A (PHYA)* resulted in delayed bud maturation and dormancy in *Populus* (Olsen et al., 1997; Rhode et al., 2002; Böhlenius et al., 2006). In transgenic poplar over-expressing an *Avena sativa PHYA*, transgenic and wild type plants ceased growth and formed terminal buds under SD; however, only the wild type became dormant (Ruonala et al., 2008). It was suggested that dormancy development was not determined by signals from the leaf, but was dependent on apical and rib meristem properties. In ethylene insensitive transgenic birch, SD did not induce terminal bud set; however, the ethylene-insensitive trees did become dormant. These studies indicate that growth cessation and terminal bud set are distinct developmental events, and that dormancy is a separate developmental process (Rinne et al., 2001; Rhode et al., 2002; Ruonala et al., 2006). Transgenic and natural mutants that are disrupted in terminal bud set, coupled with natural temporal studies, highlight the role of SD in cessation of stem elongation, terminal bud set and dormancy development. However, the mechanisms specific to the signaling cascade resulting in tissue dormancy still need clarification.

**Figure 1:**
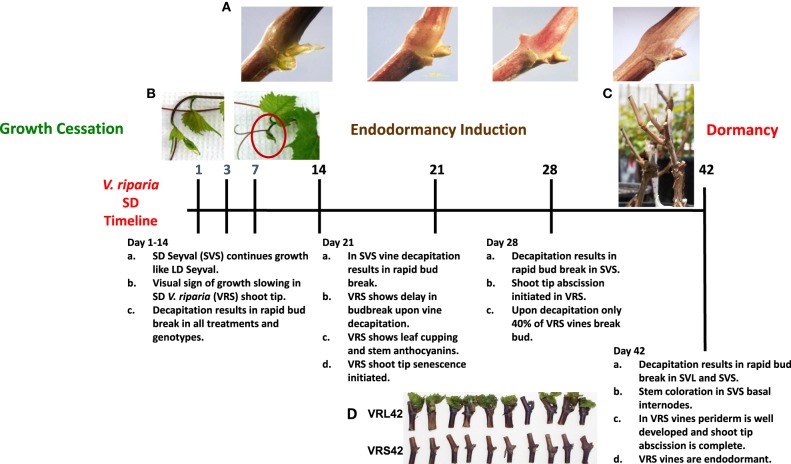
**Short photoperiod dormancy development timeline in *V. riparia* with reference to Seyval's lack of dormancy development under short photoperiods**. **(A)** Overwintering latent buds from short day (SD) treatment show budscale development and periderm development; **(B)** Actively growing *V. riparia* shoot tip from long day (LD) vines and *V. riparia* SD shoot tip during growth cessation (SD14); **(C)** Decapitated and dormant SD *V. riparia* (VRS) grapevine, the photograph was taken 28 days after the decapitated SD42 vine was returned to LD-forcing conditions; **(D)** Individual nodes (3–12 from shoot base, left to right) from *V. riparia* LD or SD vines (VRL42 or VRS42), the photo was taken 28 days after placing node sections in LD forcing conditions.

The compound axillary buds of the seasonally indeterminate grapevine provide an alternative model to explore bud dormancy. In contrast to many of the tree model systems commonly used to study dormancy development, grapevines do not set terminal buds, rather, the shoot tip abscises in response to decreasing photoperiod and/or low temperature (Fennell and Mathiason, 2002). The grapevine produces compound axillary buds containing primary, secondary and tertiary meristems throughout the growing season. The grapevine compound axillary buds remain paradormant during the growing season and will break and grow if the apical portion of the shoot is damaged or removed. The primary and secondary meristems typically contain vegetative and inflorescence primordia, whereas the tertiary meristem is predominately composed of vegetative primordia (Mullins et al., 1992). Thus, the compound axillary grapevine buds have already completed developmental processes that are associated with photoperiod induced growth cessation and dormancy in the terminal bud-forming model species (Ruttink et al., 2007; Jiménez et al., 2010; Sreekantan et al., 2010; Santamaría et al., 2011; Ueno et al., 2013). Grapevine genotypes vary in their response to SD, allowing selection of genotypes that are photoperiod responsive and non-responsive for dormancy development for dissecting the dormancy processes (Wake and Fennell, 2000; Fennell and Mathiason, 2002). Previous studies in *V. riparia* and Seyval grapevines show no differences in bud dormancy status in 14 days of long day (LD) or SD treatment, and buds in both genotypes show uncommitted tendril/floral primordia (Fennell and Hoover, 1991; Wake and Fennell, 2000; Sreekantan et al., 2010). In *V. riparia*, dormancy induction is evident after 21 days of SD by a delay in bud break, and dormancy depth increases at 28 days of SD, showing only 40% bud break. After 42 days of SD, *V. riparia* vines are dormant; neither vines nor individual nodes resume growth after 4 weeks of LD forcing conditions (Figure 1; Wake and Fennell, 2000). *V. riparia* bud water content decreases and freezing tolerance increases in SD, with a 5°C difference in freezing tolerance between LD and SD vines grown under optimal temperature conditions (Fennell and Mathiason, 2002). In contrast, Seyval is not dormant after 42 days of SD because the buds readily resume growth upon decapitation (Wake and Fennell, 2000). With these striking differences in photoperiod induced dormancy response, the grapevine system provides an excellent model system for dormancy development; therefore, this study compared global gene and key metabolite expression in *V. riparia* and *V*. hybrid Seyval. Analyses of age-matched buds from vines that were grown under LD (paradormant) or SD (dormancy induced) allowed us to dissect the molecular progress of bud dormancy. In this manuscript, dormancy refers to the endodormant phase wherein buds will not break and grow, even under favorable environmental conditions. All key terms and physiological response summary are provided in Supplementary Tables 1A,B.

## Materials and methods

### Plant materials

Potted, spur-pruned 2- to 6-year-old vines of *V. riparia* and *V*. hybrid *Seyval* were removed from cold storage and grown in LD (15 h) at 25/20 ± 3°C day/night temperatures with 600–1400 mol m^−2^ s^−1^ photosynthetic photon flux in a climate-controlled unshaded glass greenhouse (En Tech Control Systems Inc., Montrose, Minn.) in Brookings, South Dakota (44.3 N). Vines were grown in 19 L pots with shoots trained vertically. When the grapevines reached 12–15 nodes (30 days post bud break), five-vine experimental units were randomized within each of two photoperiod treatments with the same temperature and light intensity as initial growing conditions.

### Photoperiod treatments

A split plot design was used to provide LD and SD treatments (15 h and 13 h, respectively). Forty two experimental units were randomized into each photoperiod plot to provide replicates for each time point and treatment. The SD treatment was imposed using an automated white-covered black-out system (735 ft^2^ × 12 ft ceiling height; Van Rijn Enterprises LTD; Grassie, Ontario). Each photoperiod treatment (735 ft^2^) contained 42 experimental units (two genotypes × seven time points × three replicates) and spare experimental units. Five days after randomization of the experimental units (35 days post bud break) the SD photoperiod treatment was started in the SD plot and LD continued in the LD plot. Buds were harvested from each five-vine experimental unit into liquid nitrogen in separate tubes for each experimental unit. All experimental units were harvested between 8:30 and 11:30 a.m. at 1, 3, 7, 14, 21, 28, and 42 days of the LD or SD treatments. The buds were harvested from nodes 3 to 12, from the shoot base, for the entire experimental unit. Treatments were conducted between May and June with three replicate experimental units for each treatment combination in each of two consecutive years, providing six replicates in total.

### RNA extraction

Total RNA was extracted using a modified method of Chang et al. (1993). DNA was removed by incubation with 1 unit per microgram (μg) RNase-free DNase (Promega, Madison WI) at 37°C for 30 min. RNA was purified using RNeasy plant mini columns (Qiagen, Valencia CA). RNA quality and quantity were verified with an Agilent (Santa Clara, CA) 2100 Bioanalyzer RNA 6000 nano chip.

### Microarray data acquisition and analysis

Messenger RNA was converted to cDNA using reverse transcriptase and oligo dT primers containing a T7 RNA polymerase promoter sequence. Biotinylated complementary RNAs (cRNAs) were synthesized *in vitro* using T7 RNA polymerase in the presence of biotin-labeled UTP/CTP, purified, fragmented and hybridized with the GeneChip® *Vitis vinifera* (Grape) Genome Array ver. 1.0 cartridge (Affymetrix®, Santa Clara, CA). The hybridized arrays were washed and stained with streptavidin phycoerythrin and biotinylated anti-streptavidin antibody using an Affymetix Fluidics Station 400. Microarrays were scanned using a Hewlett-Packard GeneArray® Scanner and image data were collected and processed on a GeneChip® workstation using Affymetrix® GCOS software.

Expression data were first subjected to a series of rigorous quality control steps to ensure data reproducibility and overall quality following protocols previously described (Cramer et al., 2007; Tattersall et al., 2007). Average background and noise metrics were examined for consistency across all 168 arrays, as indicated by the Affymetrix GeneChip® Operating Software Users Guide. Probesets with less than 10% present calls across all arrays and Affymetrix control probesets were excluded. Raw intensity values of the remaining probesets were processed by Robust Multi-Array Average (RMA) (Irizarry et al., 2003) using the R package affy (Gautier et al., 2004). After pre-processing and normalization, a batch effect correction was conducted on all 168 arrays: to adjust for the batch effect stemming from two different years of sampling and array processing, an empirical Bayes method robust to outliers in small sample sizes was used (Johnson et al., 2007). This decreased the number of probesets with a significant batch effect from 10,773 to none. Expression data across years were combined after adjusting for batch effect, resulting in an experimental design with six replicates for each of the 28 (genotype × photoperiod × time point) treatment combinations. An additional quality control step was performed on each set of replicates: First, for each probeset, any set of six replicates having a coefficient of variation greater than 0.28 and one outlier more than 1.55 standard deviations from the mean across the six replicates was examined closely. The one outlying data point was deleted in these 3674 sets of replicates (1% of all sets of replicates). Second, any set of replicates with a notably large coefficient of variation (CV > 0.475) was completely excluded from further analyses (1% of all sets of replicates in the experiment). This yielded a dataset of 13,587 probesets with 98.8% of all expression data retained. This additional quality control step reduced the average coefficient of variation across replicates to 0.15. We found that these thresholds allowed us to identify gross outlying individual data points within replicates (Miller et al., 2009; Aw et al., 2010; Kuhn et al., 2010; Altick et al., 2012). Analyses were performed on these quality-controlled expression data of 13,587 probesets. Microarray data have been deposited in PlexDB (http://plexdb.org, VV18).

Principal component analysis (PCA) was applied to quality-controlled expression data using the correlation matrix to visualize any trends in the expression data (Gordon, 1999; Baldi and Hatfield, 2002; Knudsen, 2002; Stekel, 2003).

A Three-way ANOVA and false discovery rate (FDR; Benjamini and Hochberg, 1995) was used to test for significant main and interaction effects. The following standard model was used for this analysis: *y*_*ijkl*_ = *G*_*il*_+ *P*_*jl*_+ *T*_*kl*_+ *GP*_*ijl*_+ *GT*_*ikl*_+ *PT*_*jkl*_+ *GPT*_*ijkl*_+ ε_*ijkl*_, where *y*_*ijkl*_ denotes the log2 gene expression value (signal) measured for genotype *i*, photoperiod *j*, time point *k*, and biological replicate l, with 1 ≤ *I* ≤ 2, 1 ≤ *j* ≤ 2, 1 ≤ *k* ≤ 7, and 1 ≤ *l* ≤ 6. The terms *G*_*i*_, *P*_*j*_, and *T*_*k*_ represent the main effects of genotype, photoperiod, and time point, respectively; the terms *GP*_*ij*_*, GT*_*ik*_, and *PT*_*jk*_ represent the two-way interactions between genotype and photoperiod, genotype and time point, and photoperiod and time point, respectively. The term *GPT*_*ijk*_ represents the effect of the three-way interaction between genotype, photoperiod and time point as described in Kerr et al. (2000). There were 6331 probesets with a significant two-way photoperiod × time effect (FDR adjusted *p*-value for the two-way interaction effect *p* < 0.05). These were examined by a *post-hoc* Tukey′s Honest Significant Difference Test (HSD) with an adjustment for multiple comparisons for statistically significant photoperiod effects at each time point. Statistical significance was defined by an FDR adjusted *p*-value (*p* < 0.05). This analysis allowed examination of the significant expression between the age-matched LD and SD buds, and resulted in 3185 probesets that were differentially expressed (DE) between LD and SD at the same time point. Many probesets were DE at more than one time point. Data for all DE Photo_Time_Probesets are included in Supplementary Table 2A with a column indicating probesets that map to the same gene (Grimplet et al., 2012).

Array results were verified using real-time PCR of three replicates of RNA for five genes exhibiting different expression patterns across all time points as described in (Sreekantan et al., 2010). These genes referred to *EARLY LIGHT INDUCIBLE PROTEIN* (*ELIP1*), *HISTONE H3, STRESS ENHANCED PROTEIN 2* (*SEP2*), *PHOSPHENOLPYRUVATE CARBOXYKINASE* (*PEPCK*), and *INDOEACETIC ACID-INDUCED PROTEIN 6* (*IAA6*) (Supplementary Table 3).

All DE probesets were annotated using the *Vitis* manual curation and gene annotation from Grimplet et al. (2012; Additional File 2.2). The functional categories (MIPS) were classified using the MIPS categorization for GeneChip® *Vitis vinifera* (Grape) Genome Array in Plexdb (http://www.plexdb.org/modules/PD_probeset/annotation.php?genechip=Grape, Cramer manual curation). There are multiple probesets for some *V. vinifera* (12X V1 assembly) genes represented on the Affymetrix GeneChip® 16K *Vitis vinifera* (Grape) Genome Array ver. 1.0 (11,249 unique gene identifiers, Grimplet et al., 2012). After annotation of probesets with the *Vitis* gene annotation, multiple probesets associated with a single gene were collapsed to one unique representative (Unique ID) to determine the (1) number of unique DEGs at a time point or (2) percent of genes in 11 major functional categories for each time point (Photo_Time_DEGs, Supplementary Figure 1).

The Three-way ANOVA identified 2365 probesets with a significant three-way genotype × photoperiod × time effect (Geno_Photo_Time_Probesets, Supplementary Table 2B). Statistical significance was defined by an FDR adjusted *p*-value (*p* < 0.05). These Geno_Photo_Time_Probesets were also examined by a *post-hoc* Tukey's HSD test with adjustment for multiple comparisons for statistically significant photoperiod effects between *V. riparia* LD and SD (VRL and VRS; Supplementary Table 2C: VR_Photo_Time_Probesets) and between Seyval LD and SD (SVL and SVS; Supplementary Table 2D: SV_Photo_Time_Probesets) at each time point. These datasets in Supplementary Table 2 include all significant Geno_Photo_Time_Probesets, with a column indicating multiple probesets that map to same Unique ID. The DE probesets or genes are those that were DE between photoperiods. The DEGs are identified as up-regulated or down-regulated in SD relative to the LD expression level for their respective genotype and time point.

The genes specific to three phases of short day induced dormancy in *V. riparia* were determined by excluding from the VR_Photo_Time_Probesets, those that were also found in the SV_Photo_Time_Probesets (Supplementary Tables 2C,D). Multiple probesets associated with a single gene were collapsed to one unique representative (Unique ID) to determine (1) the number of unique DEGs at a time point (each dormancy phase DEG was counted only once in a specific phase); however, a Unique ID may occur in more than one phase (Table 1, VR_Phase_Specific_DEGs; Supplementary Tables 4A–C; VR_Perception_Phase_DEGs, VR_Induction_Phase_DEGs, VR_Dormancy_Phase_DEGs).

**Table 1:** **Number of differentially expressed genes (DEGs) categorized in perception, induction and dormancy phases of grapevine**.

**Phase**	**VR_Photo_Time_Probesets**	**SV_Photo_Time_Probesets**	**VR_Phase_Specific_DEGs**
Perception	359	344	238
Induction	493	252	461
Dormancy	1317	167	1006

*Phases are defined as: perception (D01, D03, and D07), induction (D14 and D21) and dormancy (D28 and D42). Values are VR_Photo_Time_Probesets and SV_Photo_Time_Probesets identified from post-hoc Tukey's HSD test of significant three-way interaction effects. (Supplementary Tables 2C,D). The number of DEGs specific to dormancy phases in V. riparia (VR_Phase_Specific_DEGs) and not in common with Seyval (SV) (Supplementary Tables 4A–C). Individual probesets may occur in more than one phase*.

### Gene set enrichment analysis

Gene Set Enrichment Analysis (GSEA) was conducted using GSEA-P 2.0 (http://www.broad.mit.edu/GSEA) on the quality-controlled expression values of 13,587 probesets and all VitisNet molecular networks including at least 10 genes (Supplementary Table 5A; Subramanian et al., 2005, 2007; Grimplet et al., 2009, 2012). The recommended GSEA-P 2.0 default parameters of 1000 permutations, false discovery *q*-value (*q* < 0.25) and nominal *p*-value (*p* < 0.05) were used to discover enriched molecular networks during dormancy development (Subramanian et al., 2007). Significant enrichments in VitisNet molecular networks were determined using pairwise comparisons of VRS and VRL and SVS and SVL at each time point. The significant enriched networks in common to both genotypes at the same time point were excluded since Seyval does not go dormant in response to the SD.

### Dormancy gene set comparisons

Comparisons of VR_Phase_Specific_DEGs were made with dormancy related gene lists from other dormancy gene expression studies to discover potential dormancy candidate genes. The dormancy studies included: axillary buds (*V. vinifera*, grapevine and *Euphorbia esula*, leafy spurge), terminal buds (*Populus*, hybrid poplar; *Castanea sativa*, chestnut and *Quercus petraea*, oak), cambium (*Populus*, hybrid poplar) and dormant seeds (*Arabidopsis thaliana*) (Supplementary Tables 6A–G; Cadman et al., 2006; Ruttink et al., 2007; Horvath et al., 2008; Resman et al., 2010; Santamaría et al., 2011; Díaz-Riquelme et al., 2012; Ueno et al., 2013). The best *Arabidopsis* match for the DEGs in each species was used for gene set comparisons. The datasets from these studies were determined using different platforms and they varied in size. Consequently, the number of cross-referenced genes varies widely with each comparison (Supplementary Table 6).

### Hormone analysis

Buds, as described in the plant materials section, were used for the quantification of abscisic acid and other related compounds using LC/MS/MS under Multiple Reaction Monitoring mode following the established method by Owen and Abrams (2009). Acquisition of the mass spectral data was performed according to Deluc et al. (2009).

#### Metabolite extraction and derivatization of the metabolites

All tissue samples were homogenized in liquid nitrogen and lyophilized while keeping them frozen throughout the freeze-drying procedure. Freeze-dried bud tissue (100 mg) was placed in a standard screw-cap-threaded glass vial. Polar metabolites were extracted with a water/chloroform protocol according to previously established procedures (Broeckling et al., 2005). The aqueous phase, after 1 h of extraction, containing 12.5 mg l^−1^ of ribitol as an internal standard, was evaporated over-night in a vacuum concentrator and the tube was then returned to the −80°C freezer until use. Polar samples were derivatized by adding 120 μl of 15 mg ml^−1^ of methoxyamine HCl in pyridine solution, were sonicated until all crystals disappeared, and then incubated at 50°C for 30 min. One hundred and twenty μl of MSTFA + 1% TMCS (Sigma-Aldrich, Inc., St. Louis, MO, USA) were added, the samples were incubated at 50°C for 30 min, and then immediately taken for analysis with a Thermo Finnigan Polaris Q230 GC-MS (Thermo Electron Corporation, Waltham, MA, USA). Derivatized samples (120 μl) were transferred to a 200-μl silanized vial insert and run at an injection split of 200:1 and 10:1 to bring the large and weak peaks to a concentration within the range of the detector. The inlet and transfer lines were held at 240°C and 320°C, respectively. Separation was achieved using a temperature program of 80°C for 3 min, and then increasing the temperature 5°C min^−1^ to 315°C, where it was held for 17 min. This was accomplished using a 60-m DB-5MS column (J&W Scientific, 0.25 mm ID, 0.25 μm film thickness) and a constant flow of 1.0 ml min^−1^. All organic acids, sugars and amino acids were verified with standards purchased from Sigma-Aldrich.

#### Metabolite data processing

Metabolites were identified in the chromatograms using the software Xcalibur (1.3; Thermo Electron Corporation). The software matched the mass spectrum in each peak against three different metabolite libraries: NIST (v2.0: http://www.nist.gov/srd/mslist.htm), Golm (T_MSRI_ID: http://gmd.mpimp-golm.mpg.de) and a Cramer Lab, University of Nevada Reno, custom-created library (V1) made from Sigma-Aldrich standards. Quantification of the area of the chromatogram peaks was determined using Xcalibur and normalized as a ratio of the area of the compound peak to the area of the ribitol internal standard. The accumulation of ABA, ABA conjugates and ABA catabolites (ABA, ABA-GE, DPA, PA, 7′OH-ABA, and Neo-PA) were compared in both genotypes (*Vitis riparia* and Seyval) under SD and LD. Metabolite significant differences were determined using Three-way ANOVA and Tukey's HSD test at *p* < 0.05 [*n* = 5, one entire replicate (28 samples) was lost during shipping].

## Results

### Transcriptome profiles showed a distinct photoperiod by time response (photo_time)

**Figure 2:**
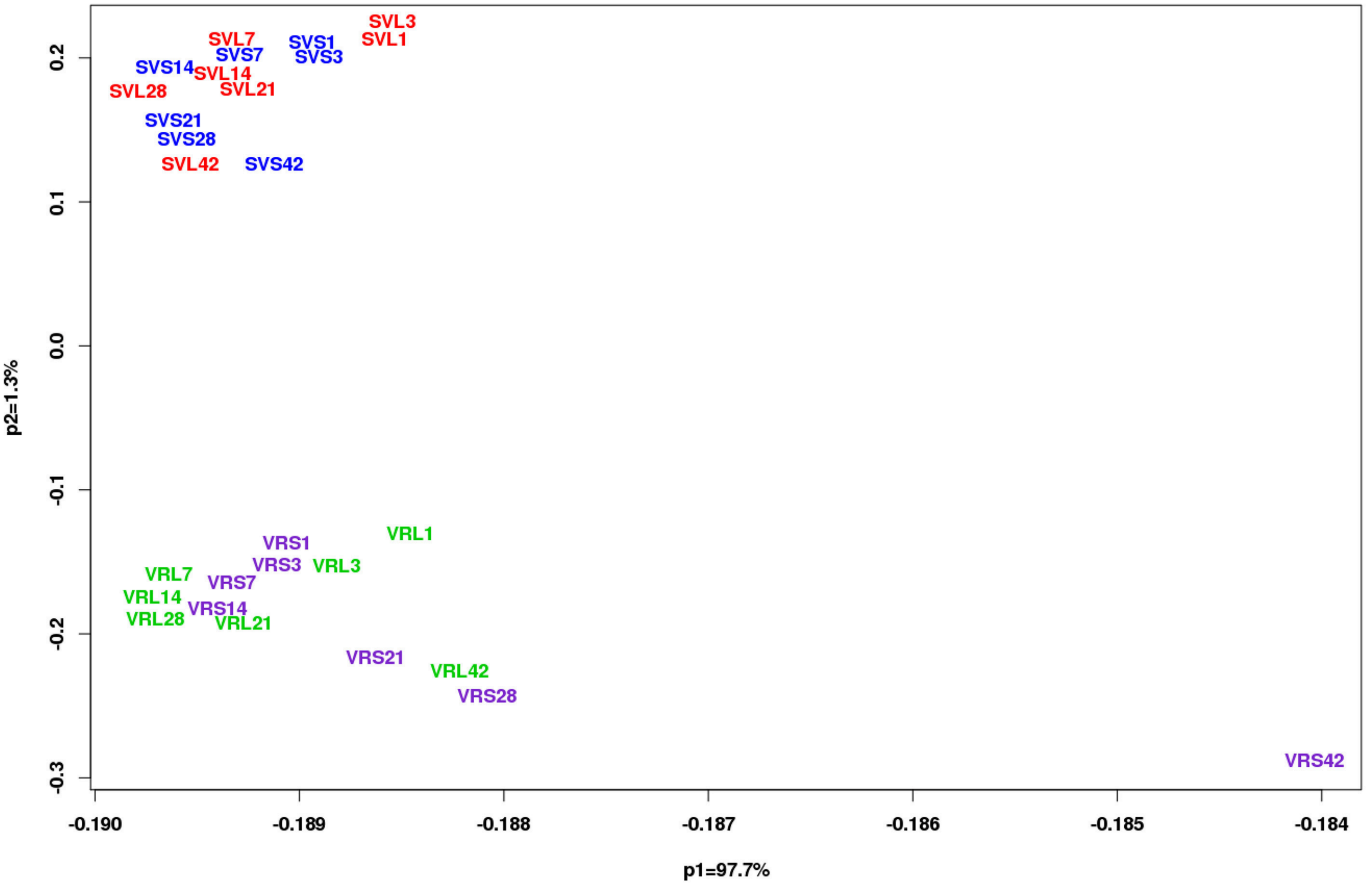
**Principal component analysis (PCA) of genotype (*V. riparia* and Seyval) and time (D01, D03, D07, D14, D21, D28, and D42 of treatment)**. Analysis was conducted on the quality-controlled data (13,587 probesets in 6 replicates) using a correlation matrix. Photoperiod treatments are color coded for each genotype. *V. riparia* [LD, green (VRL1, 3, 7, 14, 21, 28, and 42); SD, purple (VRS1, 3, 7, 14, 21, 28, and 42)] and Seyval [LD, red (SVL1, 3, 7, 14, 21, 28, and 42) and SD, blue (SVS1, 3, 7, 14, 21, 28, and 42)].

Gene expression was examined in age-matched LD buds (paradormant) and SD induced buds. The PCA showed distinct differences between the two genotypes (Figure 2). A dramatic separation of *V. riparia* gene expression under LD and SD was observed after 21 days of the photoperiod treatment. In contrast, the PCA showed limited differences in LD and SD buds across time in Seyval.

**Figure 3:**
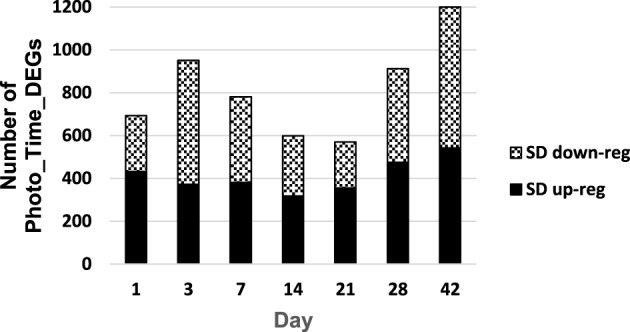
**Number of photoperiod x time differentially expressed genes (Photo_Time_DEGs) that (1) had a significant photoperiod x time interaction effect and (2) were differentially expressed between photoperiod treatments on a particular day**.

There were 3185 Photo_Time_Probesets DE in SD relative to respective LD, and some were DE at more than one time point (Supplementary Table 2A). After annotation of probesets to unique gene identifiers, the number of unique photoperiod × time differentially expressed genes (Photo_Time_DEGs) showed a bimodal pattern with the greatest differential expression at 1–7 and 28–42 days of photoperiod treatment (D01, D03, D07, D28 and D42, respectively; Supplementary Table 2A; Figure 3; Grimplet et al., 2012). The number of Photo_Time_DEGs that were DE at only one time point was most common at days 1–14 of photoperiod treatment (Supplementary Figure 1A). At D28, the majority of the Photo_Time_DEGs were also DE at D42 (Supplementary Figure 1A, Supplementary Table 2A).

The functional categories of metabolism, transcription, cellular transport, cellular communication, cell rescue/defense and unclassified genes showed a greater number of up-regulated than down-regulated genes at day one of photoperiod treatment (D01; Supplementary Figure 1B; Supplementary Table 2A). A greater number of Photo_Time_DEGs related to protein processes were up-regulated than down-regulated at day three (D03). In contrast, a greater number of Photo_Time_DEGs related to metabolism, energy and transport were down-regulated than up-regulated at day three of SD (Supplementary Figure 1C; Supplementary Table 2A). There was a greater number of Photo_Time_DEGs in functional categories of protein processes, cellular transport, and cellular communication up-regulated than down-regulated on day seven of photoperiod treatment. In contrast, there were more Photo_Time_DEGs in the categories of energy and transcription down-regulated than upregulated on day seven (D07, Supplementary Figure 1D; Supplementary Table 2A).

At 14 and 21 days of the photoperiod treatment (D14, D21), there was a greater number of Photo_Time_DEGS expressed at consecutive time points (consecutive Photo_Time_DEGs) than observed in D01 to D07. This period corresponded to the SD-induced delay in bud break in *V. riparia* (Figure 1; Supplementary Figure 1A). This period had the lowest number of Photo_Time_DEGs (Figure 3; Supplementary Figure 1A; Supplementary Table 2A). A greater number of metabolism, and protein processes genes were up-regulated than down-regulated on D14 (Figure 3; Supplementary Figure 1E). More Photo_Time_DEGs related to metabolism, cell rescue/defense, interaction with environment, developmental processes and unclassified genes were up-regulated than down-regulated on D21 of photoperiod treatment (Figure 3; Supplementary Figure 1F).

The majority of the Photo_Time_DEGs on D28 were also DE on D42 (consecutive Photo_Time_DEGs; Supplementary Figure 1A, Supplementary Table 2A). In addition, there were consecutive Photo_Time_DEGs from D21 through D42 (Supplementary Table 2A). Days 3 through 28 had a greater number of Photo_Time_DEGs down-regulated in SD in the functional category of energy (primarily photosynthesis) (Supplementary Figures 1C–H). Days 14, 21, and 28 had a greater number of DEGs up-regulated in SD relative to LD in the metabolism functional category (Supplementary Tables 2E–G). There were two peak periods with greater numbers of Photo_Time_DEGs (Figure 3). The number of Photo_Time_DEGS increased to D07, and there were fewer DEGs on D14 and D21 than on D01, D03, and D07. The number of DEGs increased again during D28 and D42. This pattern and the changing prevalence of the functional categories in relation to the physiological responses of *V. riparia*, indicated three phases of development: Perception (SD01–SD07), Induction (SD14–SD21) and Dormancy (SD28–SD42) (Figure 3).

### Differential photoperiod responses indicated three phases of dormancy development in *V. riparia*

A *post-hoc* ANOVA Tukey's HSD test identified 2365 Genotype_Photo_Time_Probesets that were DE at one or more time points during the photoperiod treatment (Supplementary Table 2B). There were a similar number of VR_Photo_Time_Probesets and SV_Photo_Time_Probesets DE at one or more time points in the perception phase (Table 1). The majority of these were DE at only one time point (Table 1; Supplementary Tables 2C,D). There was a two times greater number of VR_Photo_Time_Probesets DE than SV_Photo_Time_Probesets DE during the induction phase (days 14 and 21) (Table 1). In addition, over half the VR_Photo_Time_Probesets were DE at both 14 and 21 days, and a greater number were up-regulated than down-regulated (Supplementary Table 2C). In contrast, in Seyval, there was only one consecutive DE probeset in the induction phase. The majority of the DE SV_Photo_Time_Probesets occurred on day 14 (Supplementary Table 2D). The dormancy phase (Days 28 and 42) had the greatest number VR_Photo_Time_Probesets, and one-third of these were consecutively DE (Supplementary Table 2C). The rest of the dormancy phase DE VR_Photo_Time_probesets were DE at only one time point, with 80% of these DE on D42. This increased number occurred when the vines became unable to break bud upon decapitation. In contrast, there were fewer DE SV_Photo_Time_Probesets at these time points, and only four were DE at consecutive time points.

**Figure 4:**
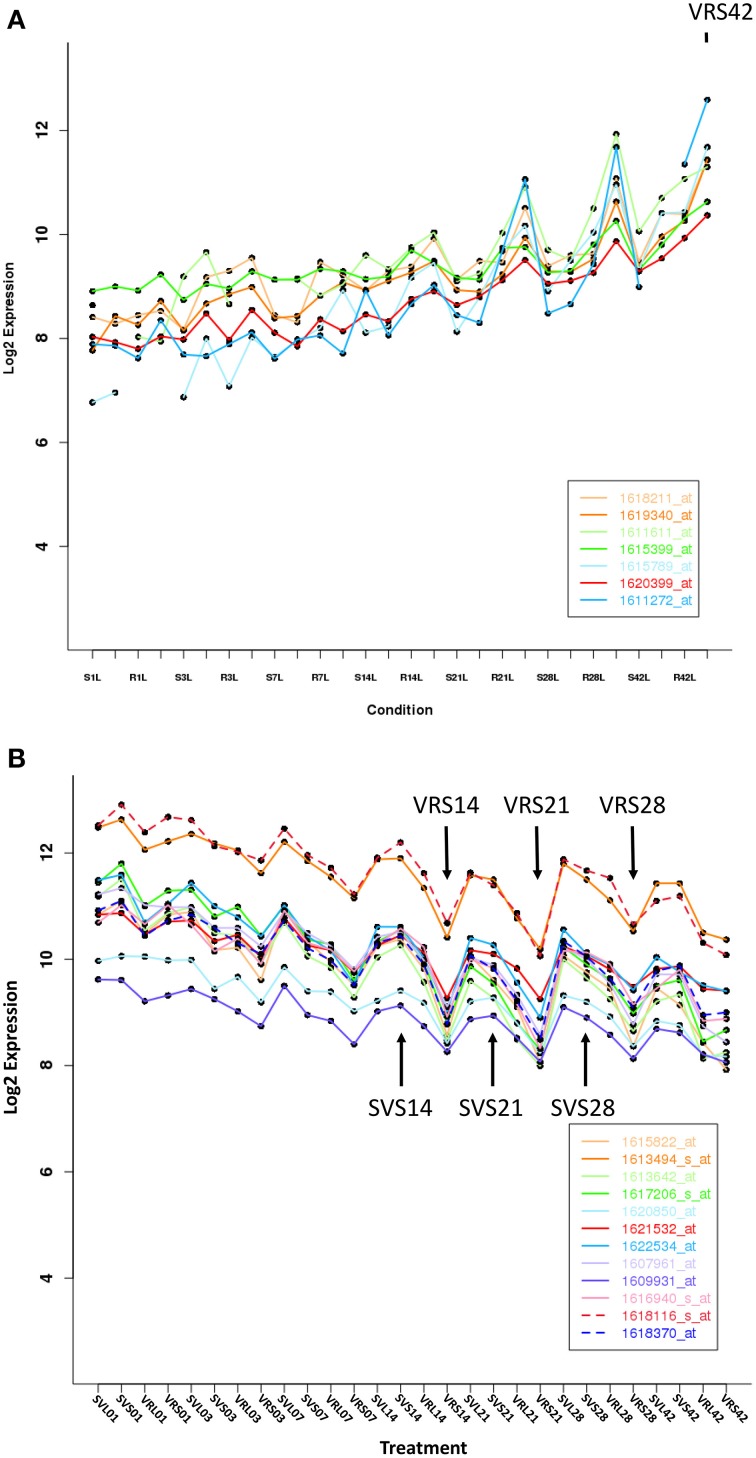
**Expression patterns for representative genes that are significantly up- or down-regulated at one or more time point in *V. riparia* (VR) or Seyval (SV)**. **(A)** Up-regulation gene expression pattern in response to short day treatment (VRS, SVS). **(B)** Down-regulation expression pattern in response to short day treatment. Expression values are log2-transformed means across replicates after normalization (*n* = 6). Arrows indicate VRS and SVS expression responses that were not similar during dormancy induction.

In the VR_Perception_Phase_DEGs, genes associated with the functional categories of response to environment (hormone signaling and cell rescue/defense responses), as well as other unknown/unclassified genes were prominent (Supplementary Table 4A). A large number of metabolism-related genes were expressed during the induction phase. For example, there were 30% metabolism-related genes up-regulated, including fatty acid biosynthesis, polysaccharide biosynthesis and degradation, phenylpropanoid biosynthesis and auxin signaling (Supplementary Table 4B). Down-regulated VR_Phase_Specific_DEGs were involved in photosynthesis and energy-related processes. Typical up- and down-regulation patterns of *V. riparia* in comparison to Seyval during dormancy development are presented in Figures 4A,B. In the VR_Dormancy_Phase_DEGs, there were equal numbers of up- and down-regulated genes, with up-regulated DEGs in the functional categories involving amino acid biosynthesis, transcription, protein processes and response to environment (hormone signaling and defense responses) (Supplementary Table 4C). Two major functional categories down-regulated in VR_Dormancy_Phase_DEGs under SD were photosynthesis/energy related and cellular communication. These data indicated a major reprogramming in *V. riparia* metabolism during the induction phase in response to a 13 h SD that continues through dormancy. It further highlights the absence of induction in Seyval in response to the SD.

### Gene set enrichment analysis (GSEA-P 2.0) distinguished potential molecular network signatures for dormancy development

Pairwise comparisons were conducted for VRS vs. VRL and SVS vs. SVL, and VitisNet molecular networks with a statistically significant over-representation of genes up-regulated in SD vs. LD at each time point in *V. riparia* and Seyval were identified (Supplementary Tables 5B,C, respectively). A total of 128 enriched networks (*SD* = 55 and *LD* = 73) were identified in *V. riparia* and 114 enriched networks (*SD* = 31 and *LD* = 73) were identified in Seyval. Molecular networks enriched in LD represent networks active during paradormancy. Molecular networks with significant over-representation of genes up-regulated in SD relative to LD in both *V. riparia* and Seyval were removed from the VRS list. Therefore, the networks significantly enriched in VRS alone were used to guide exploration of DEGs in the dormancy phases. Briefly, the perception phase showed significant enrichment in ZF-C3HC4, WRKY, and NAC transcription factor family molecular networks. The induction phase was characterized by significant enrichment in molecular networks associated with cell wall, starch and sugar metabolism and phenylpropanoid biosynthesis. Finally, the dormancy phase showed significant enrichment in molecular networks associated with amino acid metabolism and RNA and protein processing.

### Dormancy gene expression profiles in diverse species and tissues pinpointed potential dormancy candidate genes

Phase specific genes up-regulated in the *V. riparia* were compared with DEGs from Tempranillo compound axillary bud and showed two *C3HC4-TYPE RING FINGER (ZF-C3HC4)*, a *NAC DOMAIN CONTAINING PROTEIN 19* and *RESVERATROL SYNTHASE* were expressed in both *Vitis* species (Supplementary Table 6A, Díaz-Riquelme et al., 2012). *Euphorbia esula* (leafy spurge) and *V. riparia* axillary buds both had auxin signaling genes and an aquaporin gene up-regulated during dormancy (Supplementary Table 6B, Horvath et al., 2008). A comparison of poplar terminal bud and cambium tissue DEGs with *V. riparia* identified the largest number of DEGs in common between species, including genes associated with carbohydrate degradation, secondary product biosynthesis (phenylpropanoids), protein processes (folding, transport) and rescue/defense related categories. Several phenylpropanoid pathway and cell wall related genes were up-regulated in both species. These included several phenylpropanoid biosynthesis, cell wall synthesis and late embryogenesis abundant protein genes (Supplementary Tables 6C,F; Ruttink et al., 2007; Resman et al., 2010). Although, the *RESVERATROL SYNTHASE* gene was not DE in the poplar terminal buds, a *STILBENE SYNTHASE* gene was DE in poplar terminal bud and cambium tissue. Genes that were DE in *Arabidopsis* seed and *V. riparia*, included auxin and ABA signaling, *ZF-C3HC4* and *NAC DOMAIN CONTAINING PROTEIN 19* genes (Supplementary Table 6G). The majority of the DEGs found in the *V. riparia* and *Arabidopsis* comparison were up-regulated in *V. riparia*, and included auxin and ABA signaling genes. A three-way comparison of grape bud, hybrid poplar and *Arabidopsis* seed data sets showed ABA, ethylene (*ETHYLENE INSENSITIVE* 3, *EIN3*) and auxin signaling (*DORMANCY/AUXIN ASSOCIATED, DRM1*) genes and *Em PROTEIN GEA6* DE in all three species during dormancy. The differential expression of these genes in different tissue types (axillary bud, cambium and seed embryo) indicates a strong potential role in dormancy development.

### Abscisic acid, raffinose, trehalose, and resveratrol metabolites increased during dormancy

**Figure 5:**
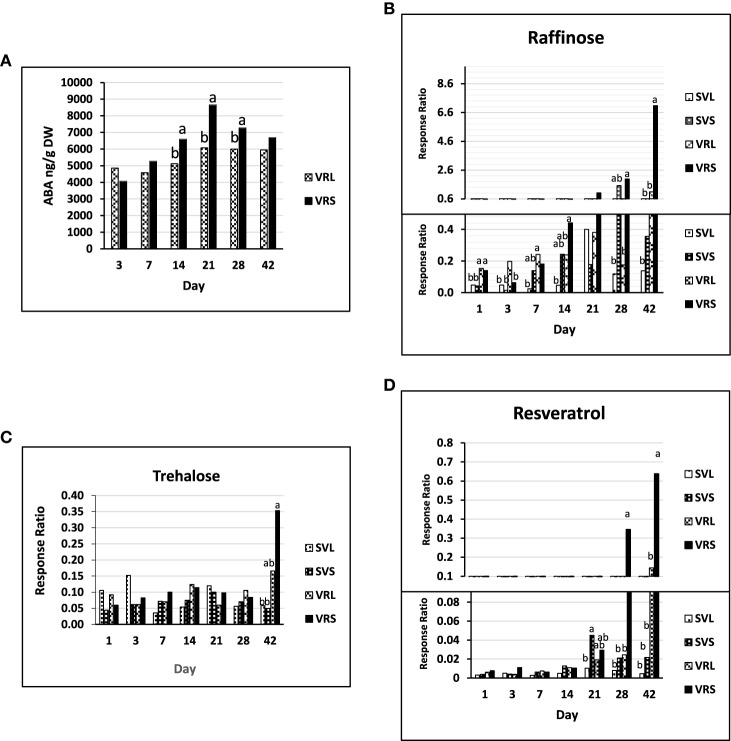
**Metabolites in *V. riparia* and Seyval under long or short day conditions (VRL or VRS and SVL or SVS)**. **(A)** Abscisic acid concentration in *V. riparia* long or short day buds (VRL or VRS, respectively). Values are ABA concentration per gram dry weight (DW) **(B)** Raffinose accumulation relative to ribitol in *V. riparia* and Seyval long or short day buds (VRL, VRS, SVL, SVS; respectively). **(C)** Trehalose accumulation relative to ribitol (response ratio) in VRL, VRS, SVL and SVS. **(D)** Resveratrol accumulation relative to ribitol (response ratio) in VRL, VRS, SVL, and SVS. Values are mean and standard error of abundance. Tukey's HSD significant differences (*p* < 0.05, *n* = 5) between SVL, SVS, VRL, and VRS at a given time point are noted by different letters, no letter indicates no difference found at that time point.

ABA significantly accumulated from day 3 until day 21, at which time it peaked in the VRL and VRS treatments (Figure 5A). The ABA concentration was significantly greater from SD14 to SD28 relative to LD, with the largest differences observed at day 21. Analyses of the conjugate form of ABA (ABA-GE) did not show any significant trend over time or between treatments, which might indicate a weak implication of conjugation events in changing the ABA levels during dormancy (Supplementary Table 7A).

A clear differential trend in glucose, fructose and sucrose concentrations was not observed (Supplementary Table 7B). In contrast, there were changes in polysaccharides known to contribute to freezing tolerance and cold acclimation. There was little raffinose accumulation during the perception phase, but there was increased accumulation during induction phase under SD with >2-fold increase from VRS28 to VRS42 (Figure 5B, Supplementary Table 7B). A similar trend was found in *V. riparia* with trehalose, with an increasing trend between VRS28 and VRS42 (2-fold, Figure 5C). The stilbenoid, resveratrol, increased from day 21 until day 42 in a similar manner as found for raffinose. This accumulation was most pronounced in *V. riparia*, with greater abundance in VRS42 than VRL42 (>3-fold, Figure 5D).

In general, there was no difference in the accumulation of the organic acids between the LD and SD in either genotype. Glycerate, malate, succinate, ribonate, and glucarate all decreased in both LD and SD in *V. riparia* and Seyval during bud development (Supplementary Table 7B). The amino acids also had similar patterns of decreasing concentrations. However, in VRS, there was an increase in leucine at 21 days, but with increasing age, the leucine levels became equivalent. In contrast, proline showed a bimodal increase, with an increased concentration in the samples VRS21 and VRS42.

## Discussion

Sensitivity to environmental cues and the ability to acclimate or become dormant to withstand stressful conditions is a hallmark of perennial plants. In widely distributed woody species, response to the reliably constant annual photoperiod cycle is used to regulate the growth cycle by initiating dormancy and acclimation processes; therefore, contributing to low winter temperature survival (Cooke et al., 2012). While much work in woody plants has focused on growth cessation and terminal bud development in response to SD, axillary buds are also subject to dormancy cycles. In the seasonally indeterminate grapevine, shoot tips abscise rather than set terminal buds, and dormancy development in the compound axillary bud is the critical factor for seasonal cycling and winter survival. The grapevine latent axillary bud develops continuously during the growing season, initiates floral meristems prior to the onset of decreasing photoperiod or low temperature cues, and is maintained in a paradormant state until either shoot apical dominance is disrupted or dormancy is initiated. In *Vitis* species, dormancy is induced by SDs and/or low temperatures (Figure 1, Fennell and Hoover, 1991; Schnabel and Wample, 1987). This differential response provides the ability to compare genotypes that are photoperiod responsive and nonresponsive for dormancy development. This removes the confounding effects of temperature, allowing one to characterize the early perception and dormancy commitment cascade resulting in the dormant state. Previous studies have shown that *V. riparia* has a 13 h critical photoperiod and Seyval has an 11 h critical photoperiod for growth cessation (Wake and Fennell, 2000). However, Seyval requires a synergistic low temperature and photoperiod interaction for dormancy induction (Garris et al., 2009). Therefore, only a 2 h photoperiod change was used in this study. *V. riparia* responds to SD (13 h) at normal growing temperatures by decreasing water content, increasing freezing tolerance, abscising the shoot tip and developing dormancy in latent axillary buds prior to leaf abscission (Figure 1, Supplementary Table 1B; Fennell and Hoover, 1991; Wake and Fennell, 2000). In contrast, Seyval slows growth and shows only a 2°C increase in freezing tolerance, and the buds remain paradormant under SD. These distinct genotype differences provided the ability to separate metabolite and gene expression changes as a general photoperiod response or a dormancy specific response. Because Seyval does not become dormant under 13 h photoperiod, genes that are DE in both genotypes were considered a general response to change in photoperiod. Thus, the DEGs in common to both genotypes were removed to create the *V. riparia* dormancy phase specific gene sets (Table 1). The remainder of the discussion focuses on the SD induced dormancy-specific responses in *V. riparia*.

### Short photoperiod, dormancy-specific responses characterized three phases of dormancy development

#### Perception phase

Differential gene expression in response to the 2 h difference in photoperiod provided evidence for a perception phase (D01, D03, and D07). The majority of differentially expressed probesets were different at only one of the time points during this phase (VR_Photo_Time_Probesets, Supplementary Table 2C). The number of differentially expressed probesets decreased during the 7-day perception phase, and there was a striking increase in the number of differentially expressed probesets during the induction phase (VR_Photo_Time_Probesets, Table 1). This suggested a pattern of perception and photoperiod entrainment, followed by a downstream trend of sustained gene activity, resulting in dormancy in *V. riparia*. The GSEA of the perception phase indicated that SD resulted in (1) down-regulation of genes associated with protein processes and fatty acid biosynthesis and (2) up-regulation of genes associated with transcription factors (ZF-C3HC4, WRKY, and NAC), sugars, and amino acid metabolism, and cytokinin signaling networks (Supplementary Table 5A). The perception phase showed multiple fatty acid biosynthesis and transport and protein processing and transport genes down-regulated in SD (Supplementary Table 4A). Sugars have been recognized as signaling molecules in dormancy (Anderson et al., 2005; Horvath et al., 2008). There were two endochitinase and alpha-glucan, alpha-amylase and alpha-galactosidases (polysaccharide degradation) genes, up-regulated in SD during the perception phase (Supplementary Table 4A). Several are also found among the poplar bud DEGs (Supplementary Table 6C). There were multiple genes associated with glutamate, asparagine, methionine, and cysteine that were up-regulated in SD during the perception phase that were not differentially expressed during the other phases (Supplementary Tables 4A–C). This indicates the potential for amino acids to act as signaling molecules in response to photoperiod, which has been shown in abiotic stress responses (Forde and Lea, 2007; Less and Galilli, 2008).

A *WRKY65* transcription factor gene, which responds to water stress and sugar starvation, was up-regulated in VRS01 and again in VRS42 (Supplementary Table 4A; Contento et al., 2004; Wang et al., 2014). This is the first indication of a photoperiod and dormancy response for *WRKY65*. No WRKY transcription factor genes were differentially expressed in leafy spurge, poplar, chestnut or oak buds; but, this may be because those studies used greater time intervals to monitor dormancy development (Ruttink et al., 2007; Horvath et al., 2008; Santamaría et al., 2011; Ueno et al., 2013). There were multiple genes encoding zinc finger binding proteins of the ZF-C3HC4 family differentially expressed throughout dormancy development, and these were both up- and down-regulated. *ZF-C3HC4* genes were up-regulated in both the perception phase and the dormancy phase. These were also DE in poplar and chestnut bud dormancy, indicating a consistent role in SD induced dormancy (Supplementary Tables 6B–F). The ZF-C3HC4 and WRKY families of transcription factors are large families, and functional roles have been linked with biotic and abiotic stress and developmental processes. The *ZF-C3HC4* genes are differentially expressed in response to SD in multiple dormancy systems (grapevine, poplar, and chestnut buds), suggesting they have a specific role in dormancy induction (Supplementary Tables 6A,C,D).

There were four genes related to flowering that were consecutively up-regulated in the perception phase, including a *MADS-BOX PROTEIN (AGL20/SOC1)* gene (VR_Preception_Phase_DEGs, Supplementary Table 4A). Flowering time genes have been implicated in dormancy because perception of photoperiod plays a role in floral initiation and growth cessation in several woody species (Böhlenius et al., 2006). Two additional transcription factor genes *FLOWERING LOCUS C (FLC)*, a negative regulator of *AGL20*, and a *SQUAMOSA PROMOTER-BINDING PROTEIN (SBP)* were up-regulated during the perception phase (Supplementary Table 4A). Both *AGL20* and the MADS-box transcription factor gene *FLC* were up-regulated in *V. riparia* SD on D01. Furthermore, *SBP* was one of the few floral related genes up-regulated on D07. Although these genes have roles in floral transitioning, the transient differential expression of *AGL20* and its negative regulator at the same time suggest an additional role in SD perception that can contribute to dormancy.

Ethylene and auxin signaling related genes were up-regulated in response to SD in *V. riparia*. Two auxin signaling genes, *DORMANCY/AUXIN ASSOCIATED and DOMAIN REARRANGED METHYLASE (DRM1)/AUXIN ASSOCIATED* were up-regulated only on day one in SD (Supplementary Tables 2C, 4A). In kiwi axillary buds, *DRM1* is up-regulated during the entire endodormancy period, and down-regulated during bud break (Wood et al., 2013). In contrast, the *DORMANCY/AUXIN ASSOCIATED* genes were up-regulated only in VRS01, apparently serving as SD perception genes. Ethylene signaling-related genes that were up-regulated in SD were an *ETHYLENE RESPONSE FACTOR* (*AP2/EREBP*) and ethylene biosynthesis inhibiting *E8 protein, ETHYLENE INSENSITIVE3* (*EIN3*). In addition, BTB/POZ DOMAIN-CONTAINING or TRAF transcription factor genes were also up-regulated (Supplementary Table 4A). The BTB/POZ DOMAIN-CONTAINING or TRAF transcription factors can assemble with *AP2/EREBP*. These signaling genes interact with other signaling pathways, as *EIN3* is noted to be involved in sugar-mediated signaling (Chao et al., 1997; Solano et al., 1998). In poplar terminal bud set, ethylene signaling occurred after 2 weeks of SD, before ABA-mediated bud maturation (Ruttink et al., 2007). However, ethylene signaling occurred early and transiently in the grapevine axillary bud perception phase (Supplementary Tables 2C, 4A). Thus, the dormancy perception phase in *V. riparia* was characterized by a rapid and transient up-regulation of several genes that encode transcription factors, ethylene and auxin signaling proteins and floral transition proteins. The temporary nature of expression of the floral transition genes also indicates a potential dual role for these genes, possibly a recruitment of their photoperiod responsiveness to serve as SD signals for the induction phase (Böhlenius et al., 2006; Sreekantan et al., 2010).

#### Induction phase

The induction phase (D14 and D21) was characterized by an increased number of probesets differentially expressed at consecutive time points (Supplementary Table 2C). The GSEA showed an enhanced up-regulation of carbohydrate metabolism (starch/sucrose and nucleotide metabolism), protein processing and phenylpropanoid biosynthesis molecular networks. By comparison, there was an enrichment in the set of down-regulated genes related to photosynthesis/energy, floral development, and circadian rhythm.

The enrichment of these molecular networks indicated changes in metabolic programming and an enhancement of secondary metabolism. Increases in bud carbohydrates are common during seasonal progression of vegetative maturation, and changes in photoperiod have been shown to reconfigure carbohydrate metabolism (Anderson et al., 2005; Ruttink et al., 2007). It should be noted that a much smaller photoperiod difference was used in this study than in studies of poplar, peach and birch; therefore the differences in carbohydrate dynamics may be less prominent in this grapevine system (Ruonala et al., 2006; Jiménez et al., 2010; Santamaría et al., 2011). A shift in the Tempranillo grapevine carbohydrate metabolism genes was noted under natural conditions. Díaz-Riquelme et al. (2012) report an increase in expression of starch and sucrose metabolism genes during grapevine bud maturation and dormancy development under field conditions. Metabolic analysis in this study indicated that there were increases in fructose, glucose, glucose-6 phosphate, sucrose, and maltose concentrations. However, carbohydrate accumulation dynamics did not show a direct association with dormancy induction patterns (Supplementary Table 7B).

Many cell wall related genes were up-regulated during the induction phase, particularly genes encoding biosynthesis enzymes (cellulose synthases, polygalacturonases, pectate lyases, invertases, and xyloglucan endotransglucosylases) (VR_Induction_Phase_DEGs, Supplementary Table 4B). Coincidently, we also found a significant number of genes in the phenylpropanoid biosynthesis network, including genes related to lignin biosynthesis. The up- and down-regulation of genes related to transport during the induction phase was not apparent in the GSEA (Supplementary Table 5A). But a distinct suite of genes encoding transporters (potassium, sulfate, nitrate, and mitochondrial and vesicle related) and *TONOPLAST INTRINSIC PROTEIN* genes (*TIP1; TIP3*) were up-regulated in SD (Supplementary Table 4B). An aquaporin located in the vacuole membrane (TIP1) is associated with vesicle targeting (Hachez et al., 2006). In addition, the genes encoding two vesicle associated proteins and a lipid transfer protein were up-regulated, but only during the induction phase.

Several genes encoding transcription factors belonging to families ZF-C3HC4, WRKY, NAC DOMAIN CONTAINING and MYB DOMAIN CONTAINING, as well as hormone signaling genes related to cell cycle and interaction with the environment were differentially expressed during consecutive time points in the induction phase (Supplementary Tables 2C, 4B). Five zinc finger transcription factor genes (*ZF-C3HC4*) were up-regulated including a *HISTONE MONO-UBIQUITINATION* 2 (*HUB2*) gene which has a role in chromatin remodeling during seed dormancy (Liu et al., 2007). Several WRKY transcription factors are involved in seed dormancy and abiotic stress tolerance (Rushton et al., 2010; Chen et al., 2012). Separate WRKY transcription factor genes were up-regulated in SD relative to LD in each of perception, induction and dormancy phases (Supplementary Table 4), and this is the first indication of their photoperiod regulation. The MYB transcription factor gene family is large and impacts a variety of developmental processes. In this study, two MYB DOMAIN PROTEIN genes (*MYB4* and *MYB14*) were up-regulated during the induction phase. *MYB4* increases freezing tolerance in transgenic *Arabidopsis* and apple plants (Pasquali et al., 2008). *MYB14* regulates isoprenoid and flavonoid metabolism in transgenic spruce (Bedon et al., 2010; Bomal et al., 2014). Flavonoid biosynthesis genes were up-regulated in grapevine buds in response to SD, as were many genes related to fatty acid and isoprenoid metabolism (Supplementary Table 4B). The NAC transcription factor has been described in shoot and embryo meristems. More recently, many NAC domain transcription factors have been associated with drought stress, but no information previously existed on their regulation by photoperiod (Aida et al., 1997; Puranik et al., 2012; Jensen et al., 2013; You et al., 2015). Three *NAC* genes were differentially expressed. The *NAC-87* gene was also up-regulated in response to SD-induced dormancy in chestnut terminal buds, further emphasizing their potential role in photoperiod induced dormancy (Santamaría et al., 2011).

In the DE hormone signaling gene set, there are several cell cycle related genes that were up-regulated (*ALPHA-EXPANSIN PRECURSER, ALPHA-EXPANSIN 3* and *BETA-EXPANSIN*). This indicated that bud development continued during the induction phase. Similarly, a suite of hormone signaling genes were up-regulated (*SNAKIN-1, E8 PROTEIN, AUXIN RESPONSIVE SAUR29, AUXIN BINDING PROTEIN ABP19a PRECURSOR, ASSOCIATED WITH PLASMAMEMBRANE-19* (*AWPM-19*)). An increase in *AWPM-19* expression was associated with ABA-induced freezing tolerance in wheat suspension cells (Koike et al., 1997). This study showed an increased ABA accumulation during the induction of bud dormancy that peaked in VRS21, and was maintained until VRS42 in comparison with VRL21 and VRL42. Control of free ABA levels through conjugation or degradation may influence the ABA mediation of the dormancy cycle (El Kayal et al., 2011). Analysis of the genes related to ABA homeostasis showed few or no changes for genes associated with the rate limiting steps of ABA biosynthesis (9-*CIS-EPOXYCAROTENOID DIOZYGENASE*, NCED; *ZEAXANTHIN EPOXIDASE ZEP, ABA1*; and *ABA DEFICIENT 2, ABA2*). One NCED gene was up-regulated in VRS28-42, and another was down-regulated in VRS42. No changes were observed in expression for the transcript involved in the conjugation of ABA into ABA-GE, which was consistent with similar levels of ABA-GE in VRS and VRL from D14 to D42 (Supplementary Table 7A). On the other hand, there was one gene involved in ABA degradation (ABA 8′hydroxylase) for which the expression level decreased from D14 until D42 in VRS (Supplementary Table 7A). We observed a similar trend for the same gene in Seyval, but the measurement of ABA in Seyval was too variable to draw any definitive conclusion. However, it is tempting to hypothesize that ABA levels under SD may be directly related to the inactivation pathway through its degradation, rather than through its synthesis as recently proposed by Zheng et al. (2015). Several factors influence *ABA-8*′*HYDROXYLASE* gene expression, including the ABA level itself and low temperature (Krochko et al., 1998; Zhou et al., 2007). Further, investigation will be needed to ascertain the influence of photoperiod on the gene regulation of *ABA-8*′*HYDROXYLASE* during endodormancy. The sustained up-regulation of gibberellin, ABA, auxin and ethylene signaling and biosynthesis genes during the induction phase clearly indicates the potential role of these hormones in the reprogramming of bud development toward induction. However, limited coverage of the hormone biosynthesis and catabolism pathways in this study limits our ability to define distinct relationships with dormancy processes.

#### Dormancy phase

The dormancy phase was characterized by a doubling of the number of differentially expressed *V. riparia* probesets in comparison with the induction phase (Table 1). There was no significant molecular network enrichment for VRS28, but VRS42 showed significant enrichment in amino acid, protein and RNA processes networks (Supplementary Table 5B). As was noted for the induction phase, there were more genes related to transport that were up-regulated than were down-regulated (Supplementary Table 4C). Furthermore, we observed a greater number of protein and vesicular transport related genes in the dormancy phase. Protein processing, phosphorylation, folding and stabilization genes were up-regulated in the dormancy phase, and there were two times the number in the dormancy phase in comparison with the induction phase (Supplementary Tables 4B,C). This indicates the potential for increased post-transcriptional processes related to dormancy.

Starch and sucrose metabolism genes were predominantly up-regulated during the dormancy phase. Increases in bud carbohydrates are common during seasonal progression of vegetative maturation, and changes in photoperiod reconfigure carbohydrate metabolism (Anderson et al., 2005; Ruttink et al., 2007). Maltose increased through the induction phase, maintaining a greater level under SD in both *V. riparia* and Seyval (Supplementary Table 7B). Maltose has been found in all organs of dormant grapevines (Stoev et al., 1960). In the dormancy phase, raffinose and trehalose increased in *V. riparia* with two-fold greater abundance in VRS42 buds (Figures 5B,C). In poplar, maltose concentrations are low, whereas raffinose and sucrose concentrations are high during dormancy, and it was suggested that enhanced expression of *SUCROSE SYNTHASE* and *GALACTINOL SYNTHASE* could drive carbohydrate accumulation toward oligosaccharides (Ruttink et al., 2007). Similarly, four *GALACTINOL SYNTHASE* genes in this study were up-regulated in VRS relative to SVS during the dormancy phase, or throughout the SD treatment. Raffinose is associated with freezing tolerance in grapevines, and *V. riparia* vines showed 5°C increased bud freezing tolerance in response SD (Fennell and Hoover, 1991; Stushnoff et al., 1993; Hamman et al., 1996; Jones et al., 1999; Fennell and Mathiason, 2002). Trehalose accumulated in a similar pattern as did raffinose. In potatoes, enhanced levels of trehalose-6-phosphate maintain tuber dormancy (Debast et al., 2011). Trehalose accumulates in small quantities in plants, and has been proposed to be a component of a sugar signaling system involving ABA and *SNF1-RELATED KINASE 1* (*SnRK1*) (Paul et al., 2008; Sonnewald and Sonnewald, 2014). It is also possible that raffinose and trehalose serve as storage carbohydrates that are recycled into glucose at bud break.

The phenylpropanoid biosynthesis network was enriched during the dormancy phase, and *RESVERATROL SYNTHASE* and two stilbene biosynthesis genes were up-regulated. Resveratrol increased in abundance, as did raffinose during the induction and dormancy phases (Figure 5D). However, a distinct relationship between metabolite levels and genes differentially expressed in the dormancy phase was not noted. Resveratrol increases in berries in response to dehydration and with tissue maturation in native grapevine species, and has been implicated in disease resistance and hypersensitive cell death (Chang et al., 2011; Deluc et al., 2011; Degu et al., 2014). Because resveratrol can act as an antioxidant, it may play a role in oxidative stress during low temperature and other abiotic stresses (Wang et al., 2010). It is also possible that resveratrol increases with maturation and provides disease protection, rather than being specific to dormancy (Langcake and Pryce, 1976).

There were 27 transcription factor families represented in the *V. riparia* phase specific genes (VR_Phase_Specific_DEGs). Twenty of these families had genes up-regulated during the dormancy phase, and seven also had members up-regulated in the induction phase (Supplementary Tables 4B,C). Of note were genes encoding members of the MYB, NAC, and WRKY transcription factor families. The MYB14 transcription factor binds to the promoter of *STILBENE SYNTHASE* and induces expression of the encoded enzyme in resveratrol biosynthesis (Fang et al., 2014). The MYB, NAC, ZINC FINGER, and LIM domain protein transcription factors have also been associated with secondary cell wall biosynthesis, and several cell wall and lignin biosynthesis genes and members of these transcription factor families are up-regulated during the dormancy phase (Oh et al., 2003; Zhong et al., 2010). However, it appears that only the *MYB14* gene is in common with the previously detected transcription factors in other dormancy systems (Supplementary Table 6). Three additional NAC family genes, *NAC19, NAC78*, and *NAC87* were up-regulated (Supplementary Table 4C). *NAC19* is a positive regulator of ABA signaling (Jensen et al., 2013). *NAC78* and *WRKY75* were up-regulated in rice overexpressing *MYB4* and associated with increased stress tolerance (Park et al., 2010). *NAC19* and *NAC87* are also up-regulated in Tempranillo grapevine buds during the transition into dormancy (Díaz-Riquelme et al., 2012). Several ZINC FINGER transcription factor genes were up-regulated during dormancy, the best characterized of these is *DOF1*. *DOF1*, a negative regulator of seed germination, appears to integrate light and hormone signaling. In *Arabidopsis, DOF1* acts between *PHYTOCHROME INTERACTING FACTOR-LIKE 5 (PIL5)* and G*IBERELLIN 3 OXYDASE*, resulting in lower levels of gibberellin, whereas *PIL5* appears to result in increased ABA levels, thus maintaining seed dormancy (Lau and Deng, 2010). Another transcription factor gene also found in dormant seeds is *BTB/POZ DOMIAN CONTAINING PROTEIN* which encodes a TRAF transcription factor found in dormant tea and rice seed (Chen et al., 2010). A *SCARECROW TRANSCRIPTION FACTOR 14* (*SCL14*) gene and *SUPEROXIDE DISMUTASE* gene were up-regulated in the dormancy phase. *SCL14* is a key transcriptional co-activator that acts with *SUPEROXIDE DISMUTASE* and other oxygen and radical detoxification genes in reactive oxygen species detoxification functions (Farmer and Mueller, 2013).

The transcription factor gene expression patterns indicated over 73 transcription factor genes (from 33 transcription factor gene families) as photoperiod regulated. Twenty of these are indicated as potential markers for the dormancy phase. Similarly, there was a large number of transport-related genes, including two vesicular transport genes that were up-regulated specifically during the dormancy phase. There were more transport genes up-regulated than down-regulated during the dormancy phase, and a distinctly different set than were found during the induction phase. Finally, there were several hormone signaling genes (genes associated with ethylene, cytokinin, auxin, and ABA) that were up-regulated during dormancy, indicating continued hormonal regulation of processes during dormancy. A comparison of the *V. riparia* dormancy phase differentially expressed genes with the Tempranillo bud dormancy related genes showed 113 genes in common, with 84% of these showing an up- or down-regulation pattern similar to that in *V. riparia* (Supplementary Table 6). However, most of the transcription factor and hormone signaling genes specific to the *V. riparia* dormancy phase are not present in the Tempranillo data set. It is possible that activation of these occurred for only a short period during early dormancy development and, therefore, they were not detected in the Tempranillo study. Comparisons across several woody dormancy systems highlight the commonality between poplar terminal bud dormancy and grapevine axillary bud dormancy. A three way comparison between poplar, leafy spurge and *V. riparia* dormancy associated genes showed flavonoid and phenylpropanoid biosynthesis genes in common. This analysis also highlighted one lignin biosynthesis gene (*Caffeic acid O-methyltransferase*) that is common to potato, raspberry, poplar, leafy spurge, and grapevine dormancy (Horvath et al., 2008). This gene might be used as a marker gene for dormancy induction.

## Conclusions

**Figure 6:**
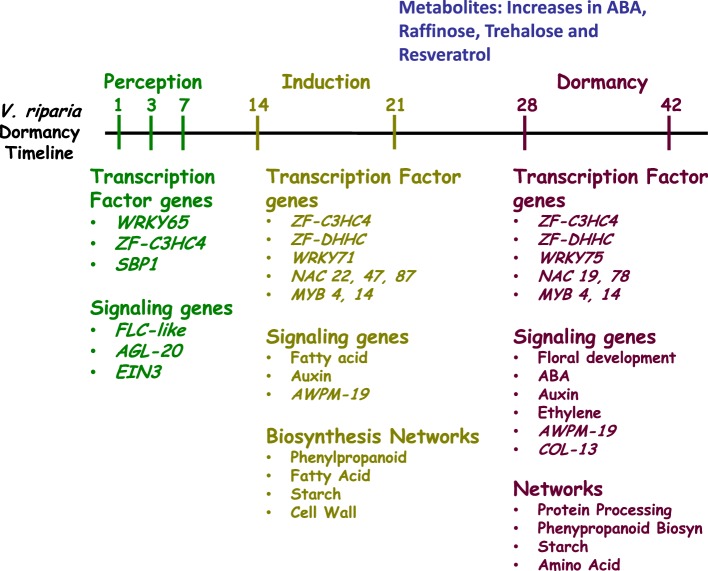
**Summary of the differentially expressed transcription factor and signaling genes and the enriched molecular networks during perception, induction or dormancy phase**. Perception phase (D01, D03, D07) is in green; induction phase (D14, D21) is in olive and dormancy phase (D28, D42) is in purple. Metabolites differentially abundant during dormancy are in blue.

Development of dormancy is a temporal and spatially regulated process in perennial species. In this study, the time frame for dormancy development in *V. riparia* was similar to that found in poplar and birch (Ruttink et al., 2007; Ruonala et al., 2008). This is striking because a smaller change in photoperiod (2 h) was used in this study, in comparison with the birch and poplar studies (6- to 8-h difference) (Ruonala et al., 2008; Ruttink et al., 2007). The similarity in time-frame indicates common photoperiod perception mechanisms between different perennial plant species. However, differences in downstream metabolite pools are likely a result of the larger photoperiod differences used in other studies (Ruttink et al., 2007). Furthermore, the 13 h photoperiod, and differential response type of the *V. riparia* and Seyval grapevines used in this study, allowed the separation of general photoperiod responses from those involved in dormancy development. Analysis of the dynamics of gene expression distinguished three phases: perception, induction and dormancy. Each phase was characterized by a distinct set of differentially expressed genes, with the dormancy phase containing the most. The induction and dormancy phases are characterized by different transcription factor and transporter sets of differentially expressed genes (Figure 6), including the NAC and WRKY transcription factor families, which have been commonly associated with water and salt stresses. The transitions from SD perception to dormancy indicate that *NAC22, NAC47, NAC87*, and *WRKY71* are characteristic of induction phase, whereas *NAC19, NAC78*, and *WRKY75* are characteristic of the dormancy phase (Supplementary Table 4). During induction, many changes in gene expression can be associated with known changes in cell walls, carbohydrates and hormone signaling, that have also been associated with cold acclimation. Finally, four metabolites (ABA, raffinose, trehalose, and resveratrol) increased in concert with the transition to dormancy in *V. riparia*. While raffinose plays a role in freezing tolerance, an important characteristic of dormant overwintering buds, the specific role of trehalose and resveratrol remains to be studied. It is interesting that dormancy induction in *Populus* promotes an up-regulation of resveratrol synthase, but whether this is a photoperiodic response or a response to reduced water content in buds is not known. The distinct phases of dormancy development and photoperiod response characteristics distinguished in this study provide a framework for dissecting both early and downstream dormancy processes and interacting components.

## Author contributions

AF, GRC, and KAS developed the experimental design. AF, SG, LGD, KM and LS conducted experiments. KAS, AF, JG, GRC, LGD, SG, and VK contributed to data analysis.

### Conflict of interest statement

The authors declare that the research was conducted in the absence of any commercial or financial relationships that could be construed as a potential conflict of interest.

## Abbreviations

ABA: Abscisic acid
ABAGE: ABA glucose ester
CV: coefficient of variation
D: day of treatment
DE: differentially expressed
DEG: differentially expressed gene
DPA: diphaseic acid
FDR: false discovery rate
GSEA: gene set enrichment analysis
LT: lethal bud freezing temperature
LD or SD respectively: long day or short day
PCA: Principal components analysis
VRL or VRS respectively: *Vitis riparia* (VR) long or short photoperiod treatment
SVL or SVS respectively, Seyval (SV) long or short photoperiod treatment. VR_Photo_Time_Probesets: Significant *V. riparia* photoperiod x time probesets
SV_Photo_Time_Probesets: Significant Seyval photoperiod x time interaction probesets
Geno_Photo_Time_Probesets: Signficant genotype x photoperiod x time probesets
VR_Phase_Specific_DEGs: *V. riparia* dormancy phase specific differentially expressed genes
VR_Perception_Phase_DEGs, VR_Induction_Phase_DEGs, or VR_Dormancy_Phase_DEGs, *V. riparia* perception: induction or dormancy phase specific differentially expressed genes.
