# Supplementary material for: Short day transcriptomic programming during induction of dormancy in grapevine
Source: Front Plant Sci. 2015 Nov 4;6:834. doi: 10.3389/fpls.2015.00834 (PMC4632279; doi:10.3389/fpls.2015.00834)
Supplement: Supplementary file 8 [file Presentation1.PPTX]

## Slide 1
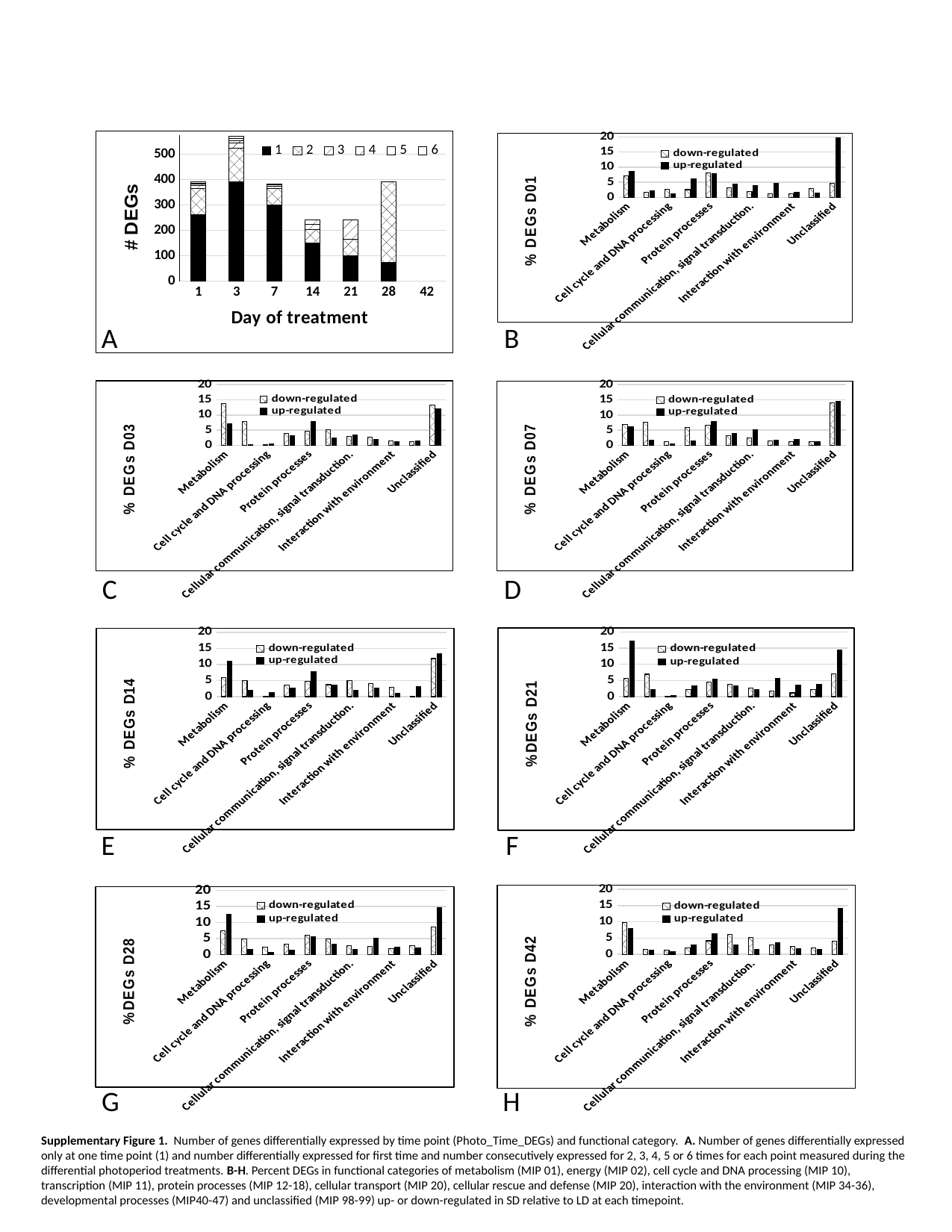

### Chart
| Category | 1 | 2 | 3 | 4 | 5 | 6 |
|---|---|---|---|---|---|---|
| 1 | 261.0 | 105.0 | 10.0 | 7.0 | 1.0 | 6.0 |
| 3 | 390.0 | 134.0 | 21.0 | 9.0 | 9.0 | 6.0 |
| 7 | 301.0 | 64.0 | 10.0 | 4.0 | 2.0 | None |
| 14 | 149.0 | 53.0 | 23.0 | 15.0 | None | None |
| 21 | 100.0 | 66.0 | 75.0 | None | None | None |
| 28 | 74.0 | 316.0 | None | None | None | None |
| 42 | None | None | None | None | None | None |
### Chart
| Category | down-regulated | up-regulated |
|---|---|---|
| Metabolism | 7.131537242472266 | 8.55784469096672 |
| Energy | 1.7432646592709984 | 2.0602218700475436 |
| Cell cycle and DNA processing | 2.694136291600634 | 1.109350237717908 |
| Transcription | 2.535657686212361 | 6.022187004754358 |
| Protein processes | 8.082408874801903 | 7.765451664025356 |
| Cellular transport, transport facilitation | 3.1695721077654517 | 4.27892234548336 |
| Cellular communication, signal transduction. | 1.9017432646592711 | 3.9619651347068143 |
| Cell rescue, defense | 1.109350237717908 | 4.754358161648177 |
| Interaction with environment | 1.2678288431061806 | 1.5847860538827259 |
| Developmental Processes | 3.011093502377179 | 1.4263074484944533 |
| Unclassified | 4.595879556259905 | 19.6513470681458 |A
B
### Chart
| Category | down-regulated | up-regulated |
|---|---|---|
| Metabolism | 13.856812933025402 | 7.043879907621248 |
| Energy | 7.967667436489608 | 0.3464203233256351 |
| Cell cycle and DNA processing | 0.3464203233256351 | 0.5773672055427251 |
| Transcription | 4.041570438799076 | 3.117782909930716 |
| Protein processes | 4.618937644341801 | 7.852193995381063 |
| Cellular transport, transport facilitation | 5.080831408775981 | 2.5404157043879905 |
| Cellular communication, signal transduction. | 2.886836027713626 | 3.4642032332563506 |
| Cell rescue, defense | 2.771362586605081 | 1.9630484988452657 |
| Interaction with environment | 1.3856812933025404 | 1.1547344110854503 |
| Developmental Processes | 1.2702078521939952 | 1.3856812933025404 |
| Unclassified | 13.279445727482678 | 12.009237875288683 |
### Chart
| Category | down-regulated | up-regulated |
|---|---|---|
| Metabolism | 6.827880512091039 | 6.116642958748222 |
| Energy | 7.681365576102419 | 1.7069701280227598 |
| Cell cycle and DNA processing | 1.1379800853485065 | 0.5689900426742532 |
| Transcription | 5.974395448079658 | 1.5647226173541962 |
| Protein processes | 6.685633001422476 | 7.823613086770982 |
| Cellular transport, transport facilitation | 3.1294452347083923 | 3.982930298719772 |
| Cellular communication, signal transduction. | 2.418207681365576 | 5.120910384068279 |
| Cell rescue, defense | 1.422475106685633 | 1.7069701280227598 |
| Interaction with environment | 1.2802275960170697 | 1.991465149359886 |
| Developmental Processes | 1.1379800853485065 | 1.2802275960170697 |
| Unclassified | 14.082503556187767 | 14.65149359886202 |C
D
### Chart
| Category | down-regulated | up-regulated |
|---|---|---|
| Metabolism | 5.758157389635317 | 17.274472168905948 |
| Energy | 6.90978886756238 | 2.3032629558541267 |
| Cell cycle and DNA processing | 0.19193857965451055 | 0.3838771593090211 |
| Transcription | 2.111324376199616 | 3.262955854126679 |
| Protein processes | 4.414587332053743 | 5.3742802303262955 |
| Cellular transport, transport facilitation | 3.8387715930902107 | 3.262955854126679 |
| Cellular communication, signal transduction. | 2.6871401151631478 | 2.111324376199616 |
| Cell rescue, defense | 1.727447216890595 | 5.566218809980806 |
| Interaction with environment | 1.1516314779270633 | 3.45489443378119 |
| Developmental Processes | 2.111324376199616 | 3.8387715930902107 |
| Unclassified | 7.1017274472168905 | 14.39539347408829 |
### Chart
| Category | down-regulated | up-regulated |
|---|---|---|
| Metabolism | 5.914972273567468 | 11.090573012939002 |
| Energy | 4.990757855822551 | 2.033271719038817 |
| Cell cycle and DNA processing | 0.18484288354898337 | 1.2939001848428837 |
| Transcription | 3.512014787430684 | 2.5878003696857674 |
| Protein processes | 4.805914972273567 | 7.763401109057301 |
| Cellular transport, transport facilitation | 3.6968576709796674 | 3.512014787430684 |
| Cellular communication, signal transduction. | 4.990757855822551 | 2.033271719038817 |
| Cell rescue, defense | 4.066543438077634 | 2.7726432532347505 |
| Interaction with environment | 2.957486136783734 | 1.1090573012939002 |
| Developmental Processes | 0.18484288354898337 | 3.1423290203327174 |
| Unclassified | 11.829944547134936 | 13.308687615526802 |E
F
### Chart
| Category | down-regulated | up-regulated |
|---|---|---|
| Metabolism | 9.84643179765131 | 8.039747064137307 |
| Energy | 1.5356820234869015 | 1.3550135501355014 |
| Cell cycle and DNA processing | 1.3550135501355014 | 0.8130081300813009 |
| Transcription | 2.077687443541102 | 2.890695573622403 |
| Protein processes | 4.155374887082204 | 6.233062330623306 |
| Cellular transport, transport facilitation | 6.142728093947606 | 2.9810298102981028 |
| Cellular communication, signal transduction. | 5.239385727190606 | 1.5356820234869015 |
| Cell rescue, defense | 2.9810298102981028 | 3.6133694670280034 |
| Interaction with environment | 2.4390243902439024 | 1.6260162601626018 |
| Developmental Processes | 2.077687443541102 | 1.4453477868112015 |
| Unclassified | 4.0650406504065035 | 14.182475158084914 |
### Chart
| Category | down-regulated | up-regulated |
|---|---|---|
| Metabolism | 7.434052757793765 | 12.470023980815348 |
| Energy | 4.916067146282973 | 1.6786570743405276 |
| Cell cycle and DNA processing | 2.278177458033573 | 0.7194244604316548 |
| Transcription | 3.357314148681055 | 1.4388489208633095 |
| Protein processes | 6.115107913669065 | 5.635491606714628 |
| Cellular transport, transport facilitation | 4.796163069544365 | 3.357314148681055 |
| Cellular communication, signal transduction. | 2.7577937649880093 | 1.5587529976019185 |
| Cell rescue, defense | 2.6378896882494005 | 5.0359712230215825 |
| Interaction with environment | 1.9184652278177456 | 2.278177458033573 |
| Developmental Processes | 2.7577937649880093 | 2.038369304556355 |
| Unclassified | 8.513189448441247 | 14.628297362110313 |G
H
Supplementary Figure 1. Number of genes differentially expressed by time point (Photo_Time_DEGs) and functional category. A. Number of genes differentially expressed only at one time point (1) and number differentially expressed for first time and number consecutively expressed for 2, 3, 4, 5 or 6 times for each point measured during the differential photoperiod treatments. B-H. Percent DEGs in functional categories of metabolism (MIP 01), energy (MIP 02), cell cycle and DNA processing (MIP 10), transcription (MIP 11), protein processes (MIP 12-18), cellular transport (MIP 20), cellular rescue and defense (MIP 20), interaction with the environment (MIP 34-36), developmental processes (MIP40-47) and unclassified (MIP 98-99) up- or down-regulated in SD relative to LD at each timepoint.
